# Pumpkin seeds, lemongrass essential oil and ripleaf leaves as feed additives for *Ascaridia galli* infected laying hens

**DOI:** 10.1007/s11259-022-10042-5

**Published:** 2022-11-30

**Authors:** Anna L. Rodenbücher, Michael Walkenhorst, Mirjam Holinger, Erika Perler, Zivile Amsler-Kepalaite, Caroline F. Frey, Meike Mevissen, Veronika Maurer

**Affiliations:** 1 Research Institute of Organic Agriculture (FiBL), 5070 Frick, Switzerland; 2 Vetsuisse-Faculty, Department of Infectious Diseases and Pathobiology, Institute of Parasitology, University of Bern, 3012 Bern, Switzerland; 3 Vetsuisse-Faculty, Department of Clinical Research and Veterinary Public Health, Division Veterinary Pharmacology and Toxicology, University of Bern, 3012 Bern, Switzerland

**Keywords:** Helminths, Medicinal plant, *Cymbopogon citratus*, *Curcurbita pepo*, *Plantago lanceolate*

## Abstract

The present study was performed to evaluate the *in vivo* efficiency of *Curcurbita pepo* (pumpkin) seeds, *Cymbopogon citratus* (lemongrass) essential oil and *Plantago lanceolata* (ripleaf) leaves against helminth infections in laying hens. In the first experiment, 75 Lohmann LSL Classic hens naturally infected with *Ascaridia galli* were assigned to groups of five; groups were randomly assigned to one of three treatments with five replicates each (untreated control; lemongrass oil: 1 g/bird/day; pumpkin seeds: 10 g/bird/day). Feed consumption and egg production were continuously recorded, individual faecal egg counts were determined weekly, and *E. coli* and *Lactobacillus* spp. three times during the experimental period of 29 days. After slaughter, intestinal worms were counted and sexed. Pumpkin improved feed conversion as compared to the control (*p* = 0.008) and to lemongrass (*p* = 0.021); no treatment effect on any other parameter was found.

In the second experiment, 75 LSL pullets were artificially infected with 3 × 200 *A. galli* eggs, randomly divided into groups of five and assigned to one of three treatments (untreated control, lemongrass oil: 1 g/bird/day; ripleaf: 5% of ration). After 109 days of sampling as described above, hens were slaughtered and worm burdens determined. Performance of the animals did not change regardless of the treatment and none of the treatments resulted in changes of the microbiological and parasitological parameters. In conclusion, with the exception of improved feed conversion in the pumpkin group, no positive nor negative effects of the additives on performance, parasitological and microbiological parameters of naturally and artificially *A. galli* infected laying hens were observed.

## Introduction

Infections by helminths in laying hens are prevalent worldwide. In a study carried out in eight European countries (AT, BE, DK, DE, IT, NL, SE, UK) between 2012 and 2014, *Ascaridia galli* was the most common parasite on organic layer farms with a prevalence of 69.5% (Thapa et al. 2015). Infections with this parasite cause various symptoms such as loss of appetite and lower locomotion activity (Gauly et al. 2007), reduced muscular development and increased mortality. Furthermore, they can reduce nutrient utilization and thereby lower the weight gain in laying hens (Sharma et al. 2019). Helminths may also increase the risk of secondary infections with bacteria, e.g. *Pasteurella multocida* and *Escherichia coli* (Dahl et al. 2002; Permin et al. 2006). Ascaridiosis was demonstrated to increase feed consumption, shorten the time of ground pecking, lower the movement and prolong nesting times, whereas no significant change of feather pecking was found (Gauly et al. 2007).

Laying hens are therefore routinely treated with anthelmintics, regardless if it is an organic farm or not (Bestman and Wagenaar 2014). The active substances flubendazole and fenbendazole are registered for use in egg producing hens with a withdrawal period of zero days. However, according to the organic regulation No. 2018/848 (EC 2018), eggs cannot be marketed as organic during and 48 h after anthelmintic treatment. This measure is intended to reduce anthelmintic residues in eggs, as well as reduce adverse enviromental impacts and effects on invertebrates in the entire food chain (Jean-Pierre and Errouissi 2002; Wagil et al. 2014).

In addition, there is a risk of developing resistances against synthetic anthelmintics. Recently, Collins et al. (2019) identified resistance to fenbendazole in *Ascaridia dissimilis*, the most common intestinal helminth of turkeys. Efforts to develop alternatives for synthetic antiparasitic drugs are therefore growing, especially in the organic sector (Hoste et al. 2013).

Many medicinal plants e.g. green tea (*Camellia sinensis* L) are known for their beneficial effects on growth-rate, feed consumption, immune system and blood composition in poultry via their immunostimulatory properties, as has been discussed in detail by Hashemi and Davoodi (2012), Khan et al. (2012) and Pliego et al. (2020). The addition of turmeric and thyme powder resulted in a higher live weight gain and higher feed intake especially in the group fed with both plants in broiler (Fallah and Mirzaei 2016). A high effectiveness on performance, feed intake and feed conversion rate of antibacterial, prebiotic and antiprotozoal acting medicinal plants was observed in poultry infected with gastrointestinal bacteria or protozoa as is presented in detail in the review by Farinacci et al. (2022).

In the last years, medicinal plants such as *Carica papaya* (papaya), *Curcuma longa* (curcuma), *Zingiber officinale (*ginger), *Punica granatum* (pomegranate) and *Azadirachta indica* (neem) were considered as natural antiparasitics in poultry. *In vitro* experiments showed mortality rates of adult or larval *A. galli, Heterakis* spp., and *Capillaria spp.* comparable to synthetic anthelmintics after exposure to the aqueous or ethanolic extract of the plants (Abdul Aziz et al. 2018; Alam et al. 2014; Bazh and El-Bahy 2013). Pineapple (*Ananas comosus*), neem, pomegranate, ginger and curcumin were successfully tested for their lethal effects on adult *A. galli* in artificially infected chickens, when compared to a negative and/or a positive control (Abdul Aziz et al. 2018; Bazh and El-Bahy 2013; Patra et al. 2010).

Pumpkin oil as a feed supplement was shown to decrease the concentration of blood cholesterol and triglyceride as well as the mortality of broiler chicken (Hajati 2011). *Curcurbita pepo* (pumpkin) is a fruit belonging to the family *Cucurbitaceae.* Further studies showed a high anthelmintic efficiency of pumpkin extracts on worm mortality, which was similar compared to fenbendazole in *in vitro* and *in vivo* in *A. galli* artificially infected chickens (Abdul Aziz et al. 2018). Acorda et al. (2019) observed a moderate efficiency of pumpkin seeds compared to the mebendazole treated group for reducing worm counts and faecal egg output of *A. galli, Heterakis* spp. and *Raillietina* spp. in naturally infected chickens.

*Cymbopogon citratus* (lemongrass) is a herb belonging to the *Poaceae* family. Its essential oil increased the productive performance of laying hens by its anti-inflammatory, immunomodulatory, antioxidant and antimicrobial effects (Alagawany et al. 2021). There is also evidence for its potential of increasing live weight gain in broiler chicken (Mukhtar et al. 2013) and the feed conversion and growth of juvenile quails (Alagawany et al. 2021). The use of lemongrass oil and its anthelmintic efficiency was shown in *in vivo* experiments in artificially infected gerbils (Macedo et al. 2015) and naturally infected sheep (Macedo et al. 2019).

Studies on ripleaf (*Plantago lanceolata,* family *Plantaginaceae*; also known as ribwort plantain) as a main component (90%) of a herbal mixture increased feed consumption and egg production in laying hens (Rahman et al. 2021). Using ripleaf as feed additive for broiler chickens showed a high efficiency regarding the live weight gain and feed consumption (Chowdhury et al. 2013). Ripleaf is a tannic plant (Fayera et al. 2018), which is known for its anthelmintic properties, e.g. limiting the availability of nutriments for larvae, inhibiting oxidative phosphorylation in adults and larvae, binding to intestinal larval mucosae and thereby causing autolysis (Symeonidou et al. 2018).

Pumpkin and ripleaf are accepted as feed material according to the Commission Regulation No. 2017/1017 (EC 2017). Lemongrass is registered as a feed additive in Europe according to Regulation No. 1831/2003 (EC 2003). All three are promising candidates for replacing or complementing synthetic anthelmintics in the future.

The aim of this study was to investigate the anthelminthic potential of lemongrass essential oil, pumpkin seeds and ripleaf leaves as feed components in *A. galli* infected laying hens on station. For the first time, these medicinal plants were tested both in naturally as well as in artificially infected hens in the same study setting. The hypotheses tested included the plants having the potential to (a) reduce faecal egg counts and worm burdens in naturally infected hens, (b) reduce *A. galli* establishment and faecal egg counts in artificially infected hens, and (c) impact on *in ovo* larval development without (d) having negative effects on performance, health and welfare of the hens.

## Materials and methods

### Study design and housing

The study was divided into two experiments. In the first experiment, hens naturally infected with *A. galli.* (NI) were used. In the second experiment, young hens were artificially infected with *A. galli* (AI). Both experiments were carried out at the experimental facility of the Research Institute of Organic Agriculture. Space allowance and equipment in the compartments corresponded to the Swiss Animal Welfare Ordinance and to organic standards (BioSuisse 2020) with the exception that the hens had no access to pasture or outdoor runs. All animals had *ad libitum* access to water and a standard organic layer feed. Litter was replaced twice a week and faeces under the perches were removed daily in order to prevent re-infection by *A. galli*. Between the experiments, the house was thoroughly cleaned and disinfected.

#### Experiment with naturally infected hens (NI)

The first experiment was carried out between April and May 2021. Lohmann LSL Classic white laying hybrids (*n* = 75) were bought from a commercial Swiss organic farm and brought to the experimental facility at 71 weeks of age. At arrival, each bird was individually ringed, examined, weighted and faecal samples were taken. Birds were then distributed to 15 groups based on their live weight (LW) and faecal egg count (FEC) on day 1 in order to obtain groups with similar average and range of LW and FEC to minimize variation between groups (average ± SD of group averages: LW: 1657 ± 0.024 kg, FEC: 3131 ± 956 EPG). The groups were then randomly distributed to the respective treatments: control C-NI (*n* = 5) received basic feed, L-NI received lemongrass essential oil (1 g/bird/day; *n* = 5) and P-NI received pumpkin seeds (10 g/bird/day; *n* = 5) mixed into the basic feed.

The essential lemongrass oil (citral A + B 74.0%; citronellol 1.0%, geraniol 8.0%, isoeugenol 0.5%, limonene 0.5%, linalool 1.0%) was applied to diatomaceous earth in a 20% concentration, produced by SaluVet GmbH, Bad Waldsee, Germany. To reach the targeted daily dose of 1 g/bird/day of lemongrass essential oil, 5 g/bird/day of the formulated product was given. Pumpkin seed in pharmacopoeia quality was purchased from Alfred Galke GmbH, Bad Grund, Germany.

**Fig. 1:**
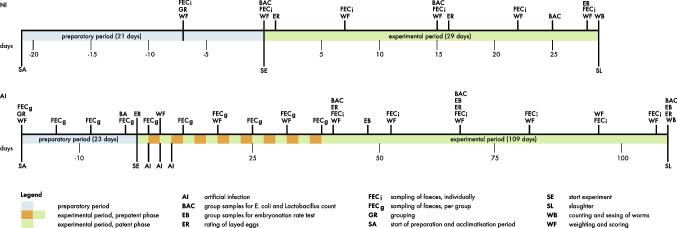
Time overview (days) of experiment with hens naturally (NI) and artificially (AI) infected with *Ascaridia galli*; NI was separated in a preparatory period (21 days) and an experimental period (29 days); AI had a preparatory period of 23 days and an experimental period of 109 days; the abbreviations are explained in the legend

After grouping, the laying hens had a preparatory period of 21 days to get accustomed to the new groups and housing conditions, (e.g. feed and environment) until the experimental feeding started (day 1). The experimental period lasted 29 days with weekly sampling, weighing and scoring. At day 29 the birds were slaughtered to count the worm burdens (Fig. 1).

#### Experiment with artificially infected hens (AI)

The second experiment was carried out between June and October 2021. Parasite naïve Lohmann LSL Classic white laying hybrids (*n* = 75) were purchased from a commercial rearing farm at the age of 17 weeks. At arrival, the hens were individually ringed and randomly distributed into 15 groups of 5 individuals. The animals were adapted to the new environment and to layer feed during 23 days until the experimental feeding started (day 1). During this period, faecal samples were analyzed weekly to confirm that animals were actually parasite naïve. The 15 groups were randomly allocated to two plant treatments and one control treatment: C-AI (*n* = 5) were fed basic feed, L-AI received lemongrass essential oil (1 g/bird/day; *n* = 5) and R-AI dried leaves of ripleaf (5% of feeding ration; *n* = 5) mixed into the basic laying hen ration.

Essential lemongrass oil was applied as described for experiment NI. The ripleaf leaves in pharmacopoeia quality were bought at Alfred Galke GmbH, Bad Grund, Germany. The hens were artificially infected at days 4, 6, 8 after onset of experimental feeding with 200 fully embryonated *A. galli* eggs per animal and infection day. *A. galli* eggs had been collected from adult females obtained from freshly slaughtered hens of the same compartment that provided hens for experiment NI and cultured according to Perler (2017).

The experimental period was 109 days with bi-weekly sampling, weighing and scoring. At the end of the experiment all birds were slaughtered and intestines were removed to count the worm burdens (Fig. 1).

### Sampling and measuring

#### Health and performance

##### Live weight and health and welfare scoring

Animals were weighed (Kern & Sohn GmbH, IFB 30K5DM, max. 30 kg) and clinically examined. Feathering and wounds were scored according to Tauson et al. (2005). Keel bone condition was palpated according to Scholz et al. (2008). The overall score with a maximum of 40 points corresponds to that of Bestman et al. (2019). These parameters were determined five times in experiment NI and nine times in experiment AI (Fig. 1).

##### Feed consumption

The birds were fed *ad libitum*. Feed consumption per group was calculated as the difference between weight of feed offered and weight of the remaining feed (Pryma Vista, kitchen scale, EK4150, max. 5 kg). Weighing took place daily in experiment NI and twice a week in experiment AI.

##### Laying performance and egg rating

Egg production was recorded daily per group. Wind eggs and broken eggs were documented and added to the total egg count. Each egg was weighed and categorized as either small (S: < 53 g), medium (M: ≥ 53 g < x < 63 g) or large (L: ≥ 63 g). At specific dates, all laid eggs per group were weighed and rated by determining the whole egg weight, the eggshell weight and the egg yolk weight (Maurer et al. 2015) as well as the color of the yolk (Yolk Fan TM, DSM, Scale: 1–16). The egg ratings took place at days 2, 16 and 28 of the experiment NI and at days 1, 40, 67 and 109 in the experiment AI (Fig. 1). The results of the last egg rating in NI and AI were taken for calculating the feed conversion rate.

#### Microbiology

At certain days (NI: days 1, 15, 28; AI: days -2, 40, 67, 109) mixed samples of freshly excreted faeces of each compartment (at least 4 g per sample) were taken and transferred to Biolytix AG, Witterswil, Switzerland for quantitative analyses of *Lactobacilli* spp*.* and *Escherichia coli* measured in colony forming units/g faeces (CFU). The methods used by the lab were ISO 15214:1998–08 (a horizontal method for counting mesophil Lactobacilli – counting of colonies at 30 °C) and DIN ISO 16649–2 (a horizontal method for the enumeration of β-glucuronidase-positive* Escherichia coli*).

#### Parasitology

##### Faecal egg count

Faecal samples were collected from individual birds isolated under small plastic boxes (40 × 29 × 17 cm^3^) for one to two hours (until defecation). Samples were analyzed using a modified McMaster technique (Schwarz et al. 2020) with a sensitivity of 50 eggs per gram faeces (EPG).

In experiment NI individual samples were analyzed once per week and in AI every second week during the patent period. Mixed faecal samples of each group were analyzed weekly during the adaptation period and the prepatent period in experiment AI to ensure parasite naïvety of the hens prior to artificial infection and to determine the end of the prepatent period (day 39) and thus the start of individual sampling (day 40; Fig. 1).

##### Embryonation rate

In experiment AI, the embryonation rate was determined in *A. galli* eggs collected from fresh faecal samples of each group at days 49, 67 and 109. Samples were washed through a sieve tower (0.5 mm, 100 μm, 32 μm) with a powerful water jet. The residue on the lowest sieve (32 μm) was transferred into a sediment tube (50 ml), restocked with water and centrifuged for five minutes at 500 g. The supernatant was removed, the tube was filled up with a sugar solution (density: 1.24 g/cm^3^) and centrifuged again for 5 min. Sieving and centrifugation were repeated with the supernatant. The suspension volume was reduced to 20 ml by a vacuum absorber and then transferred into one culture-flask per group and eggs were incubated in 0.1 N H_2_SO_4_ at 22–24 °C under dark conditions. Classification of different developmental stages of *A. galli* eggs was carried out according to Tarbiat et al. (2015) with an inverted microscope (Olympus, CK X 41 SF) after 7 and 14 days.

##### Worm burden

At the end of the trial (NI: day 29; AI: day 109), all hens were slaughtered. The intestinal tract was removed, cut open longitudinally with intestinal scissors and washed out under a light shower jet of tap water into a fine strainer (size 35 µm). The residue was flushed with a strong shower jet to separate worms from different types of remnants. *Ascaridia galli* were counted, sexed (Ackert 1931) and the juvenile stages (≤ 2 cm) were identified under a stereomicroscope for each hen individually.

### Statistical analysis

Statistical analyses were carried out using the software R (R-Core-Team 2021) by applying linear mixed effect models (function “lmer” from the package “lme4”; Bates et al. 2015) as well as simple regression models. Model specifications including fixed effects, covariables, random effects, transformations and outliers are presented in Table 1 and Table 2. Model assumptions (normal distribution of residuals and homoscedasticity) were checked through graphical analysis of model residuals. If residuals did not meet the model assumptions, a transformation of the outcome variable was applied (Table 1; Table 2). If data distribution and residuals suggested a non-linear course of a specific outcome variable, the variable “days” was fitted with natural splines with three knots. A sum contrast scheme was applied for the fixed effects and interactions. P-values were obtained by comparing the full model including all main effects and their interactions to models reduced by one main effect or interaction. The model comparison was conducted using a parametric bootstrap approach with the function “mixed” from the “afex” package (Singmann et al. 2021). Data analyses with p-values of < 0.05 were considered significant. In case of a p-value < 0.05 for a fixed effect or interaction, a post hoc test was carried out. Model estimates and confidence intervals for the full model were obtained with parametric bootstrap simulations (“predict” in the package “bootpredictlme4”; Duursma 2017).

**Table 1:** Model specifications for the outcome variables of experiment NI (natural infection)

Outcome variable	Fixed effect^a^	Covariable	Random effect	Notes
Live weight	Treatment * Time	Measurement D1	Measurement in ID in group	
Live weight gain	Treatment	None	ID in group	
Health and welfare score	Treatment * Time	Measurement D1	Measurement in ID in group	
Feed consumption	Treatment * Time	None	Measurement in group	Removal of two outliers with 77 g and 128 g feed per day (measurement error)
Total feed consumption Feed consumption per egg weight	Treatment	None	-	Simple regression models
Laying performance	Treatment * Time	None	Measurement in group	
Egg yolk color Egg yolk weight Egg weight Shell weight	Treatment * Time	Measurement D2	Egg sample in date in group	
E. coli Lactobacillus	Treatment * Time	Measurement D1	Sample in group	Log-transformation
FEC *A. galli*	Treatment * Time	Measurement D1 (log)	Measurement in ID in group	Log-transformation Two 0 values were corrected to 100 for log-transformation
Worm burden	Treatment	None	ID in group	Log-transformation of juveniles and fertility

^a^ Interactions between fixed effects are indicated with a *

**Table 2:** Model specifications for the outcome variables of experiment AI (artificial infection)

Outcome variable	Fixed effect^e^	Covariable	Random effect	Notes
Live weight	Treatment * Time	Measurement D-22	Measurement in ID in group	
Live weight gain	Treatment	Measurement D-22	ID in group	
Health and welfare score	Treatment * Time	None	Measurement in ID in group	
Feed consumption	Treatment * Time	Measurement D-3	Measurement in group	
Total feed consumption Feed consumption per egg weight	Treatment	None	-	Simple regression models
Laying performance	Treatment * Time	None	Measurement in group	
Egg yolk colour Egg yolk weight Egg weight Shell weight	Treatment * Time	Measurement D1	Egg sample in date in group	Removal of one outlier with egg shell weight of 18.3 g
E. coli Lactobacillus	Treatment * Time	Measurement D1	Sample in group	Log-transformation Removal of one outlier with E. coli count 1.5*10^8^
FEC *A. galli*	Treatment * Time	None	Measurement in ID in group	Log-transformation
Worm burden	Treatment	None	ID in group	Log-transformation of juveniles, fertility and sex ratio

^a^ Interactions between fixed effects are indicated with a *

Four individual leg rings in AI were lost during slaughtering, therefore the worm burdens of four birds (C-AI: 1 bird; L-AI: 1 bird; R-AI: 2 birds) had to be excluded from analysis. One bird of group P-NI was removed due to severely disturbed general condition not related to the trial.

## Results

### Health and performance

#### Live weight

##### Experiment NI

The live weight of the birds increased over the experimental feeding time (*p* < 0.001). No treatment dependency and no interaction of treatment and time was found. The estimated final LW was around 1680 g (Table 3).

**Table 3:** Treatments, dosages, and performance data of naturally and artificially infected hens expressed as model estimates, including 95% confidence intervals

Parameter	Treatment model estimate (lower confidence interval, upper confidence interval)			P-values
	Untreated control	Lemongrass oil (1 g/hen/day)	Pumpkin seeds (10 g/hen/day	
Performance data naturally infected hens				
Live weight on day 8 [g]	1635(1615, 1653)	1626(1609, 1649)	1641(1622, 1660)	Interaction Treatment * Time = 0.830
Live weight on day 28 [g]	1676(1656, 1696)	1682(1665, 1702)	1682(1664, 1702)	Treatment p-value = 0.560
				Time p-value < 0.001
Daily live weight gain [g]	2.0(0.95, 3.1)	2.19(1.14, 3.24)	2.19(1.14, 3.29)	Treatment p-value = 0.960
Daily feed consumption per bird [g]	142(137, 147)	136(130, 141)	123(117, 128)	Treatment *p* = 0.002
				Post Hoc Test control – lemongrass: p-value = 0.211 control – pumpkin: p-value < 0.001 lemongrass—pumpkin p-value = 0.007
Laying performance per animal on day 6	0.9(0.74, 1.04)	0.96(0.8, 1.12)	0.96(0.82, 1.12)	Interaction Treatment * Time = 0.560
Laying performance per animal on day 29	1.04(0.9, 1.2)	0.8(0.64, 0.96)	1.06(0.9, 1.2)	Treatment p-value = 0.780
				Time p-value = 0.460
Feed conversion [g feed/g egg]	2.2(2.1, 2.4)	2.2(2.0, 2.3)	1.9(1.8, 2.0)	Treatment *p* = 0.004
				Post Hoc Test control—lemongrass: p-value = 0.862 control—pumpkin: p-value = 0.008 lemongrass—pumpkin: p-value = 0.021
	Untreated control	Lemongrass oil (1 g/hen/day)	Ripleaf leaves 5% of ration	
Performance data artificially infected hens				
Live weight on day 6 [g]	1532(1503, 1560)	1513(1485, 1540)	1531(1503, 1558)	Interaction Treatment * Time = 0.003
Live weight on day 106 [g]	1675(1649, 1704)	1594(1567, 1622)	1645(1619, 1673)	Treatment p-value = 0.570
				Time p-value < 0.001
				Post Hoc Test for Day 106 control – lemongrass: p-value = 0.015 control – ripleaf: p-value = 0.362 lemongrass – ripleaf: p-value = 0.228
Daily live weight gain [g]	1.48(1.22, 1.72)	0.74(0.5, 1.00)	1.23(0.97, 1.48)	Treatment p-value = 0.01
				Post Hoc Test control – lemongrass: p-value = 0.005 control – ripleaf: p-value = 0.418 lemongrass – ripleaf: p-value = 0.077
Daily feed consumption per bird [g]	131(125, 136)	131(125, 136)	22(117, 128)	Treatment p-value = 0.04
				Post hoc Test: control—lemongrass: p-value = 1.000 control—ripleaf: p-value = 0.070 lemongrass—ripleaf: p-value = 0.080
Laying performance per animal on day 1	0.72(0.62, 0.82)	0.64(0.56, 0.74)	0.56(0.48, 0.66)	Interaction Treatment * Time = 0.400
Laying performance per animal on day 109	0.98(0.9, 1.08)	0.98(0.88, 1.08)	1.0(0.9, 1.1)	Treatment p-value = 0.090
				Time p-value < 0.001
Feed conversion [g feed/g egg]	2.6(2.4, 2.8)	2.5(2.4, 2.7)	2.5(2.3, 2.6)	Treatment = 0.60

##### Experiment AI

Live weight of young hens increased during the experiment (*p* < 0.001). The estimated final LW of the animals ranged between 1590 g in L-AI and 1680 g in C-AI at the end of the study. A treatment effect was detected (*p* = 0.010) and an interaction between treatment and time was observed (*p* = 0.003). Compared to the standard curve for Lohmann LSL Classic, the LW of animals in all treatments was above average at the beginning, but below the standard curve at the end of the experiment. A post hoc test for day 106 showed a difference between C-AI with the highest values and L-AI with the lowest values (*p* = 0.015), whereas there was no difference between the other treatments (Table 3).

#### Feed consumption

##### Experiment NI

No overall treatment effect was observed on the total feed consumption (*p* = 0.340). However, an interaction between treatment and time was detected (*p* < 0.001). Feed consumption in C-NI nearly stayed on the same level, whereas in L-NI and especially in P-NI feed consumption decreased over the experimental time (*p* < 0.001). The estimated feed consumption ranged between 141 g/day in P-NI to 149 g/day in L-NI in the beginning and between 121 g/day in P-NI and 139 g/day in C-NI at the end of the experiment. In a post hoc test for day 28, no significant differences between the respective treatments were found.

A treatment effect on the daily feed consumption was detected (*p* = 0.002); the post hoc test showed a lower average daily feed consumption in P-NI compared to L-NI and C-NI (Table 3).

##### Experiment AI

An overall treatment effect was estimated for the total feed consumption (*p* = 0.006). Overall, feed consumption increased over time (*p* < 0.001) with animals of the R-AI group always showing the lowest value. An interaction between treatment and time was observed (*p* < 0.001). Consumption of L-AI exceeded consumption of C-AI after approximately three weeks and decreased again after seven weeks into the trial. The post hoc test for day 106 revealed no significant differences between any of the treatments and control.

A treatment effect was detected for daily feed consumption (*p* = 0.040); the post hoc test showed a tendency for a lower average daily feed consumption in R-AI compared to C-AI and L-AI (Table 3).

#### Laying performance

##### Experiment NI

Hens laid numerically on average between 0.9 (in L-NI) to 0.94 (in P-NI) eggs per day. Neither an effect of treatment, of time, nor an interaction between treatment and time was confirmed (Table 3). Egg production was higher compared to the standard curve of Lohmann LSL Classic.

##### Experiment AI

Young hens laid numerically on average between 0.91 (in R-AI) to 0.94 (in L-AI) eggs per day. The laying performance of the young birds increased during the experiment (*p* < 0.001) and reached a plateau after five weeks of trial. C-AI started with a higher laying performance and R-AI with the lowest. A tendency of a treatment effect was found (*p* = 0.090), whereas an interaction between treatment and time was not found (*p* = 0.400). Compared to the standard curve of Lohmann LSL Classic, egg production was slightly above average at the beginning and in average in week eight of trial.

#### Egg rating

##### Experiment NI

No effects of treatment or treatment and time interactions were observed for total egg weight (model estimate: 68 g), shell weight (model estimate: 10 g), egg yolk weight (model estimate: 19 g) and egg yolk color. Both total egg weight and egg yolk weight increased over the experimental period and showed a time dependent effect (*p* = 0.070; *p* = 0.040). Egg yolk color got brighter on the rating scale during the experiment (*p* < 0.001).

##### Experiment AI

No effects of treatment or treatment and time interaction were observed for total egg weight (model estimate: day 40: 56 g in L-AI to 58 g in R-AI; day 109: 60 g in L-Ai to 64 g in R-AI), egg yolk weight (model estimate: day 40: 13 g; day 109: 17 g) and egg yolk color. For shell weight (model estimate: stayed between 8 and 9 g) no effect of treatment, but an interaction between treatment and time was observed (*p* = 0.260; *p* = 0.010). In a slope coefficient post hoc test the difference between L-AI and R-AI over time was significant (*p* = 0.030). R-AI showed the highest incline of the treatment, whereas L-AI-values stayed at almost the same level during the experimental period. No difference between C-AI and R-AI and between C-AI and L-AI was found (*p* = 0.098; *p* = 0.845). Total egg weight, shell weight and egg yolk weight increased over the experimental period (all: *p* < 0.001). Egg yolk color got brighter on the rating scale by around 1.5 points during the experiment (*p* < 0.001).

#### Feed conversion rate

##### Experiment NI

The model revealed a treatment effect on the feed conversion rate (*p* = 0.004). The estimated feed conversion rate of P-NI (lowest value: 1.9 g feed/g egg) differed significantly from C-NI and L-NI (both 2.2 g feed/g egg; Table 3).

##### Experiment AI

No effect of treatment was detected in the young, artificially infected animals (*p* = 0.600). The estimated feed conversion rate ranged between 2.5 and 2.6 g feed/g egg (Table 3).

#### Health and welfare scoring

##### Experiment NI

No treatment effect on the scores was observed (*p* = 0.570). Out of 40 points, all treatments scored 31 points at the beginning and approximately 32 points at the end (*p* = 0.002). An interaction between treatment and time was detected (*p* = 0.030). A slope coefficient post hoc test showed a significant discrepancy between L-NI, which remained approximately at the same level and P-NI with the highest incline (*p* = 0.040), i.e. improvement of health score. C-NI compared to L-NI and C-NI compared to P-NI were not different (*p* = 0.828; *p* = 0.151). Independently of the treatment, several birds showed diarrhea during the experiment (C-NI: *n* = 11; L-NI: *n* = 11; P-NI: *n* = 7).

##### Experiment AI

At the beginning of the study, all birds scored 40 points and approximately 38 points at the end (*p* < 0.001). An effect of treatment was not confirmed (*p* = 0.47). There was an interaction between treatment and time (*p* = 0.002). In a slope coefficient post hoc test C-AI compared to L-AI and L-AI compared to R-AI showed a difference (*p* = 0.005; *p* = 0.007), whereas C-AI and R-AI were not different (*p* = 0.217). Independently of the treatment, some hens suffered from diarrhea (C-AI: *n* = 2; L-AI: *n* = 1; R-AI: *n* = 2) and some had filthy feathering around the cloaca (C-AI: *n* = 5; L-AI: *n* = 2; R-AI: *n* = 2).

### Bacteriology

#### Experiment NI

##### *Lactobacillus spp*.

The *Lactobacillus* spp. count increased slightly over the experimental time (from 1.1 to 5.6, Mio/g faeces; *p* < 0.001). L-NI stayed almost at the same level whereas C-NI had a higher incline, followed by P-NI. Neither treatment effect, nor an interaction between treatment and time was detected (*p*= 0.240; *p* = 0.410).

*E. coli* count decreased during the experiment (from 3.3 to 0.9 Mio/g faeces; *p* < 0.001). L-NI had the lowest digression, whereas C-NI and P-NI deviated almost parallel. C-NI had the lowest *E. coli* count over the whole experimental time. Neither treatment effect, nor an interaction between treatment and time was detected (*p* = 0.600; *p* = 0.470).

#### Experiment AI

##### *Lactobacillus spp*.

The *Lactobacillus* spp. count increased over the experimental period (from 124.1 to 340.5 Mio/g faeces; *p* = 0.020). C-AI showed the highest incline. L-AI and R-AI had almost the same incline, but L-AI showed higher values during the whole experiment. Neither treatment effects nor an interaction between time and treatment was detected (*p* = 0.850; *p* = 0.790).

*E. coli* count increased over time (from 0.5 to 8.3 Mio/g faeces; *p* = 0.006). L-AI and R-AI had a high incline, whereas C-AI only increased slightly until the end. No effect of the treatment or an interaction between treatment and time was observed (*p* = 0.140; *p* = 0.440).

### Parasitology

#### Faecal egg counts

##### Experiment NI

**Fig. 2:**
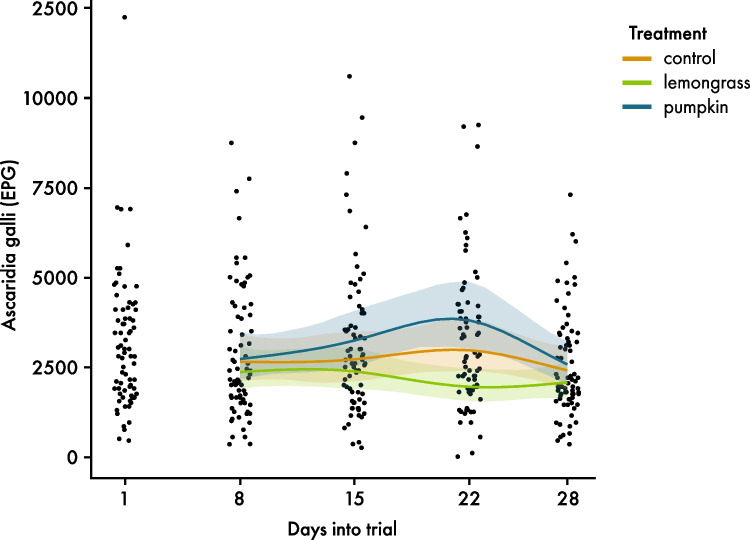
Development of faecal egg count of *Ascaridia galli* eggs per gram faeces over time (days) during the experiment with naturally infected hens

There was no effect of treatment, time nor an interaction between treatment and time on *A. galli* FEC (*p* = 0.690; *p* = 0.110; *p* = 0.230). As presented in Fig. 2, FECs were similar in all treatments at the beginning, whereas at day 22 FECs were slightly reduced in L-NI, increased in C-NI and were even higher in P-NI. At the end of the experiment, the FEC of all treatments decreased to a similar level, which was slightly lower than the starting values (Fig. 2).

##### Experiment AI

**Fig. 3:**
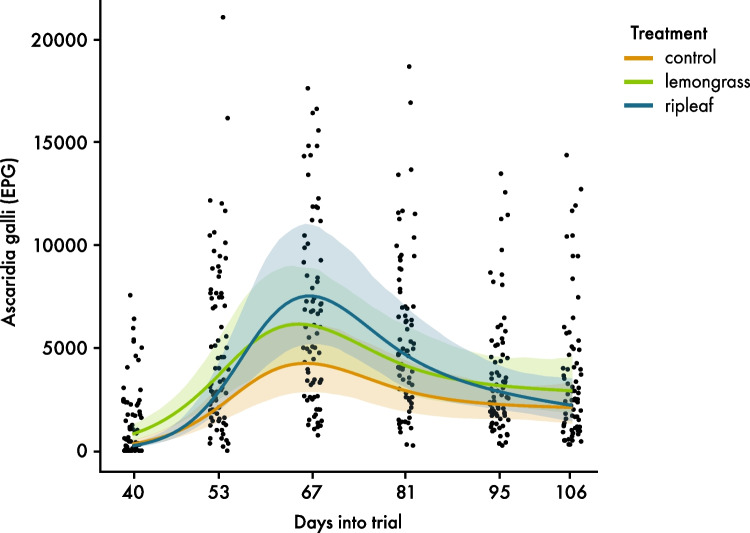
Development of faecal egg count of *Ascaridia galli* eggs per gram faeces over time (days) during the experiment with artificially infected hens

A time and a treatment-dependent effect on FEC was detected (*p* < 0.001; *p* = 0.003). FECs clearly increased in all treatments. There was a peak at day 67, which was highest in R-AI followed by L-AI and then C-AI. In all treatments, FECs decreased afterwards and were at a higher level on day 106 (median FEC: C-AI: 2.450 eggs/gram faeces; L-AI: 2.485 eggs/gram faeces; R-AI: 2.500 eggs/gram faeces) than at the beginning (median FEC: C:AI: 425 eggs/gram faeces; L-AI: 1.450 eggs/gram faeces; R-AI: 350 eggs/gram faeces). Over the complete experimental period, FECs were lowest in C-AI (Fig. 3). The interaction between treatment and time was significant (*p* = 0.005). A post hoc test for day 67 showed a tendency for a difference between C-AI compared to L-AI (*p* = 0.085), but no difference between C-AI compared to R-AI and L-AI compared to R-AI (*p* = 0.208; *p* = 0.869).

#### Embryonation rate in experiment AI

**Fig. 4:**
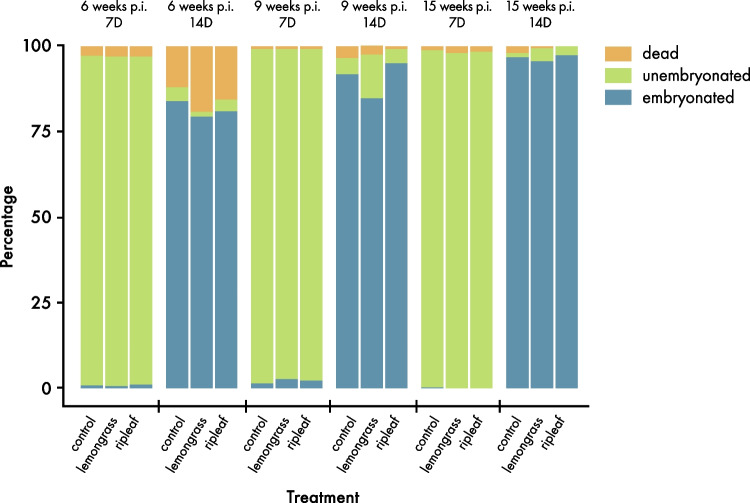
Percentage of dead, unembryonated and embryonated eggs after 7 and 14 days storage at room temperature in the experiment with artificially infected hens

After seven days of incubation, mostly unembryonated *A. galli* eggs were found (estimated: around 97%). Dead eggs were approximately two percent and embryonated eggs roughly one percent. After 14 days of incubation, 3 to 6% of the eggs were unembryonated, 6 to 8% dead and 87 to 91% embryonated (Fig. 4).

#### Worm burden

##### Experiment NI

The total worm count ranged between 8 to 111 worms per hen. The sex ratio ranged on average between between 0.7 (in P-NI) and 0.8 (in L-NI and C-NI) males to females. The proportion of juveniles was on average between 9.3% ± 8.4% (in C-NI) to 12.3% ± 12.6% (in L-NI). No treatment effects on any parameter were observed (Table 4).

**Table 4:** Worm burden (males, females, juveniles) and generated parameters as model estimates and extreme values at day of slaughter in naturally and artificially infected hens

Parameter	Treatment model estimate (lower confidence interval, upper confidence interval; minimum–maximum)			P-values
	Untreated control	Lemongrass oil (1 g/hen/day)	Pumpkin seeds (10 g/hen/day)	
Worm burden naturally infected hens				
Total	50(42, 60; 23–110)	57(48, 65; 8–98)	54(45, 63; 24–111)	Treatment p-value = 0.64
Males	19(15, 23; 9–34)	20(16, 24; 3–39)	19(15, 23; 7–56)	Treatment p-value = 0.85
Females	26(22, 31; 13–60)	29(24, 33; 5–51)	29(24, 34; 13–60)	Treatment p-value = 0.71
Juveniles	2.3(1.2, 4.3; 0–21)	4.0(2.2, 8.3; 0–24)	2.4(1.3, 4.8; 0–38)	Treatment p-value = 0.4
Fertility	74(52, 106; 16–185)	64(47, 92; 8–350)	79(54, 110; 25–477)	Treatment p-value = 0.76
	Untreated control	Lemongrass oil (1 g/hen/day)	Ripleaf leaves (5% of ration)	
Worm burden artificially infected hens				
Total	48(34, 60; 10–134)	48(34, 62; 10–152)	40(27, 55; 2–112)	Treatment p-value = 0.68
Males	22(16, 28; 4–63)	21(15, 26; 2–65)	20(13, 26; 2–59)	Treatment p-value = 0.81
Females	25(17, 32; 3–76)	26(18, 34; 3–87)	19(12, 27; 0–51)	Treatment p-value = 0.45
Juveniles	0.3(0.2, 0.6; 0–3)	0.6(0.3, 1.0; 0–7)	0.5(0.2, 0.8; 0–6)	Treatment p-value = 0.32
Fertility	117(69, 192; 10–1217)	155(96, 251; 14–1594)	147(86, 243; 10–1200)	Treatment p-value = 0.82

##### Experiment AI

The total worm count ranged between 2 to 152 worms per hen. The sex ratio ranged on average between 1.0 (in L-AI) and 1.2 (in R-AI) males to females. The establishment rate (percentage of total worm count compared to number of eggs used for infection) was on average 8%. In all treatments, the proportion of juveniles was on average between 1.7% ± 2.8% (in C-AI) and 4.3% ± 5.5% (in L-AI). No treatment effects on any parameter were found (Table 4).

## Discussion

Adding medicinal plants such as pumpkin, ripleaf and lemongrass to the feed had no impact on (a) faecal egg counts and worm burden in naturally infected hens, (b) the establishment of *A. galli* and faecal egg counts in artificially infected hens, and (c) *in ovo* larval development. However, (d) no negative effects on performance, health and welfare were observed.

The naturally infected old layers showed a slight increase of LW until the end of the trial. Normally, LW tends to remain constant at that age (Lohmann-Breeders 2021), but this was not the case in our experiment. This was probably due to the better housing conditions and smaller group size in the experimental units as compared to the situation on a commercial farm. Similar effects have been observed by Yilmaz Dikmen et al. (2016) who found a higher live weight gain in free-ranged hens compared to animals without free range access.

Lemongrass treatment resulted in a significantly lower LW gain of artificially infected birds over the whole duration of the experiment. Compared to the standard curve, the LW gain of the control group had the slightest and lemongrass the highest deviation from the standard curve. Abdelqader et al. (2007) observed no difference in LW gain between uninfected and artificially *A. galli* infected birds, indicating that the results in our study might have been caused by the dosage of lemongrass oil. This is in accordance with Tiwari et al. (2018), who tested three dosages of lemongrass oil in broiler chicken, the lowest of them at a similar dosage as in our study, and observed a lower LW gain in this group compared to the higher dosages and an untreated control group. However, the highest dosage tested by Tiwari et al. (2018) also resulted in a lower total weight gain in the end of the experiment when compared to control.

Pumpkin meal as a feed component might improve the production parameters of poultry, such as the feed conversion rate (Achilonu et al. 2018) as seen in our experiment with naturally infected birds. Lower feed consumption in the P-NI group may have been caused by higher metabolisable energy content in this feed (Ravindran et al. 2016). Because feed consumption in the P-NI group was significantly reduced, pumpkin was replaced by ripleaf in the experiment with young layers, whose feed requirements are increased due to the start of egg production and simultaneous growth (Pottgüter 2016). However, the same pattern found with pumpkin was also observed with ripleaf in the experiment with artificially infected young layers. Like in the experiment with naturally infected older layers, this had no influence on the total weight gain, which shows a tendency of a better feed conversion rate due to ripleaf supplementation. In contrast, no impact of ripleaf or other tanniferous plants on the feed conversion rate was reported in other studies (Hidayat et al. 2021; Temur and Uslu 2019).

No negative effects on performance have been associated with the medicinal plants. Especially concerning egg composition, such as shell weight, egg weight, yolk color and yolk weight, no negative influence of the feed additives was found either in the experiment with the old nor the young layers. Also, no negative consequences regarding the laying performance were observed in the experiments. In contrast, there is evidence of a positive influence of pumpkin oil on egg quality and laying performance (Adsul and Madkaikar 2021; Herkeľ et al. 2014). Studies about ripleaf as a main component showed a positive effect on egg production in laying hens (Rahman et al. 2021).

Feed conversion was lower in the experiment with artificially infected hens (2.5–2.6 g feed/g egg) than in the naturally infected old layers (1.9–2.2 g feed/g egg). Feed utilization and therefore feed conversion can be affected by parasitic infections (Collins 2021). Other studies showed similar feed conversion rates, and they are comparable to the data obtained in this study in laying hens artificially infected with *A. galli* (Sharma et al. 2018a) and naturally *A. galli* infected hens (Sharma et al. 2018b). The slightly lower feed conversion rate in the experiment with artificially infected hens might have been due to the higher worm burden or to the requirement for growth.

The health and welfare scores of naturally infected older hens remained approximately at the same level during the entire experiment. Young hens scored several times the maximum points at the beginning, but their health and welfare score regressed during the experiment. Concerning the feathering, e.g., Kjaer (2000) observed a decrease of the scores at the beginning of the study followed by a slight increase at 69 weeks of age.

World-wide, infections with *E. coli* are the cause for significant morbidity and mortality in poultry (Ewers et al. 2003; Nakazato et al. 2009). They may cause multiple organ lesions and also pericarditis, peritonitis, airsacculitis, perihepatitis, salpingitis and other extra-intestinal diseases (Ewers et al. 2003; Kathayat et al. 2021). Permin et al. (2006) revealed a negative effect on body weight gain and found a tendency for increased mortality in animals infected with both, *A. galli* and *E. coli*. In both our experiments no influence of the feed additives on the *E. coli* count was found. Essential oils are known for their antimicrobial potential (Dušan et al. 2006; Murbach Teles Andrade et al. 2014). Bölükbaşi et al. (2008) showed a decrease of the *E. coli* count in faeces of laying hens by adding the essential oils of *Thymus vulgaris* (thyme), *Rosmarinus officinalis* (rosemary) and *Salvia sclarea* (sage) in their basal diet.

In both experiments, NI and AI, no treatment-based effect on the count of *Lactobacillus* spp. was found. *Lactobacillus* spp. are the majority of indigenous bacteria inhabiting the intestinal tract of poultry on the first day (Harimurti and Hadisaputro 2015). *Lactobacillus* spp. may suppress the growth of pathogens, probably by secreting antibacterial components like peroxides, bacteriocins and lactic acids and are therefore crucial for pathogen control in the microflora and for the immune system in poultry (Harimurti and Hadisaputro 2015). Pliego et al. (2020) described that plants such as *Camellia sinensis* (green tea) and *Zingiber officinale* (ginger) increased *Lactobacillus* spp. count.

No effect of any plant component on FEC was observed in the two experiments. This was unexpected, since all plants included in the trials and in particular lemongrass essential oil significantly reduced survival of *A. galli* females in previous *in vitro* assays (Maurer et al. unpublished results). One reason for the discrepancy between the *in vitro* and the *in vivo* data might be due to the dose and the concentrations reached in the organism, especially the gut, compared to the concentration obtained *in vitro*. The concentration *in vitro* is likely to be much higher compared to the final concentration in the small intestine *in vivo*. Tiwari et al. (2018) evaluated lemongrass oil as a growth promotor in chicken by dosing up to 3.6 g/bird/day. No negative consequences were observed at this dose, which indicates that we could have increased the dosage of lemongrass oil by 3.6 times. Also, a great portion of lemongrass essential oil might have been absorbed already before the duodenum and thus, the concentration in the small intestine might have been too low for killing the worms and resulting in a lower EPC.

In addition to its effects on adult worms, ripleaf was shown to affect embryonation and survival of *A. galli* eggs *in vitro* (Maurer et al. unpublished results). By observing the embryonation rate in cultivated *A. galli* eggs, we tried to reveal differences between the groups, to get a better understanding of their influences on the development of worm eggs. Since there was no significant outcome, it can be concluded that lemongrass oil and ripleaf do not have any influence on the development of the worm eggs at the dosages used in this study. An inhibition of development by using plant extracts was shown in several *in vitro* studies, but *in vivo* experiments are not commonly done. A high efficiency of *Aloe secundiflora* crude extracts was demonstrated by inhibiting the development of *A. galli* eggs *in vitro* (Kaingu et al. 2013). Stephen et al. (2022) examined *Sterospermum kunthianum* (Cham-Holl) leaf extracts and their influence on *in vitro A. galli* egg development and found a concentration-dependency of the plant extract. Like ripleaf (Fayera et al. 2018), Cham-Holl and *A. secundiflora* have tanniferous components which are known for their anthelmintic properties such as limiting the availability of nutrients for larvae, inhibiting oxidative phosphorylation in adults and larvae, binding to intestinal larval mucosae and causing thereby autolysis (Symeonidou et al. 2018). Sen et al. (2020) investigated different extracts of *Carica papaya* (papaya) *in vitro* and artificially *A. galli* infected animals *in vivo* and showed its high efficiency on larval development *in vitro* and its FEC reducing abilities *in vivo*. However, a treatment effect on the embryonation rate in *in vivo* was not observed in this study. In the future, it should be considered to investigate the embryonation rate *in vivo* with tanniferous plants. Having no outcome by using ripleaf in the *in vivo* experiment, it should be considered to test different crude extracts and different dosages.

Pumpkin, lemongrass oil and ripleaf were promising candidates for replacing or complementing synthetic anthelmintics in the future: Abdul Aziz et al. (2018) showed a similar effect of pumpkin seed ethanolic extract compared to fenbendazole, which increased over treatment time. A moderate efficiency compared to mebendazole for reducing worm counts and faecal egg output of *A. galli* was observed by Acorda et al. (2019). Lemongrass oil was used as an herbal anthelmintic in *in vivo* experiments in artificially infected gerbils (Macedo et al. 2015) and naturally infected sheep (Macedo et al. 2019) with a moderate effect on the worm burden.

Tanniferous plants, such as ripleaf are known for their anthelmintic properties on the larvae and worms themselves (Symeonidou et al. 2018). Maurer et al. (unpublished results) revealed a high *in vitro* effectiveness of pumpkin seeds, ripleaf and lemongrass oil as a natural anthelmintic depending on the dosage. Possibly due to the pH and other gastrointestinal factors, the worms responded differently to the *in vivo* treatment. There was no significant outcome concerning the worm burden, such as female, male or juvenile worms, fertility, proportion of juveniles, or sex ratio in both experiments. To our knowledge, this is the first study on ripleaf and lemongrass essential oil as natural anthelmintics in laying hens, the dosage for the animals and the transfer from *in vitro* to *in vivo* experiments should be considered in further investigations. Thereby, one of the main goals should be to minimize the gastrointestinal and kinetic factors *in vivo*.

Resistance against anthelmintics has been shown for *Ascaridia dissimils* in turkeys (Collins 2021; Perkins et al. 2012) and a lowered efficiency of 85.5% was reported for fenbendazole against *A. galli* (Yazwinski et al. 2013). According to the World Association for the Advancement of Veterinary Parasitology the efficiency of an anthelmintic should be ≥ 90% (Yazwinski et al. 2003), thus alternatives are urgently needed (Hoste et al. 2013). However, other studies still reported full efficiency on *A. galli* for benzimidazoles in chickens (Feyera et al. 2022; Tarbiat et al. 2017).

Nevertheless, synergistic effects between anthelmintic and medicinal plants with anthelmintic properties should be investigated.

## Conclusion

The present study revealed no evidence of anthelmintic effects of pumpkin seeds, lemongrass oil and ripleaf leaves in naturally and artificially *A. galli* infected hens. The absence of effects on health, welfare and production parameters by any plant tested indicated that these medicinal plants are well tolerated, and can be used as feed additives. Pumpkin seed improved feed conversion and ripleaf slightly reduced feed consumption without having any negative influence on performance data, which makes both plants promising future feed components for poultry. Future studies should be performed to study higher doses of the respective medicinal plants in laying hens.

## Data Availability

The data and material that support the findings of this study are available from the corresponding author, [vm], upon reasonable request.
